# Aortic and mitral flow quantification using dynamic valve tracking and machine learning: Prospective study assessing static and dynamic plane repeatability, variability and agreement

**DOI:** 10.1177/2048004021999900

**Published:** 2021-02-27

**Authors:** Julio Garcia, Kailey Beckie, Ali F Hassanabad, Alireza Sojoudi, James A White

**Affiliations:** 1 Department of Cardiac Sciences, University of Calgary, Calgary, AB, Canada; 2 Department of Radiology, University of Calgary, Calgary, AB, Canada; 3 Stephenson Cardiac Imaging Centre, University of Calgary, AB, Canada; 4 Libin Cardiovascular Institute, University of Calgary, Calgary, AB, Canada.; 5 Alberta Children’s Hospital Research Institute, Calgary, AB, Canada; 6 Circle Cardiovascular Imaging, Advanced Technologies, Calgary, AB, Canada

**Keywords:** Bicuspid aortic valve, mitral valve, 4D-flow magnetic resonance imaging, valve tracking, machine learning

## Abstract

**Background:**

Blood flow is a crucial measurement in the assessment of heart valve disease. Time-resolved flow using magnetic resonance imaging (4 D flow MRI) can provide a comprehensive assessment of heart valve hemodynamics but it relies in manual plane analysis. In this study, we aimed to demonstrate the feasibility of automate the detection and tracking of aortic and mitral valve planes to assess blood flow from 4 D flow MRI.

**Methods:**

In this prospective study, a total of *n* = 106 subjects were enrolled: 19 patients with mitral disease, 65 aortic disease patients and 22 healthy controls. Machine learning was employed to detect aortic and mitral location and motion in a cine three-chamber plane and a perpendicular projection was co-registered to the 4 D flow MRI dataset to quantify flow volume, regurgitant fraction, and a peak velocity. Static and dynamic plane association and agreement were evaluated. Intra- and inter-observer, and scan-rescan reproducibility were also assessed.

**Results:**

Aortic regurgitant fraction was elevated in aortic valve disease patients as compared with controls and mitral valve disease patients (*p* < 0.05). Similarly, mitral regurgitant fraction was higher in mitral valve patients (*p* < 0.05). Both aortic and mitral total flow were high in aortic patients. Static and dynamic were good (r > 0.6, *p* < 0.005) for aortic total flow and peak velocity, and mitral peak velocity and regurgitant fraction. All measurements showed good inter- and intra-observer, and scan-rescan reproducibility.

**Conclusion:**

We demonstrated that aortic and mitral hemodynamics can efficiently be quantified from 4 D flow MRI using assisted valve detection with machine learning.

## Introduction

Moderate or severe valvular heart disease (VHD) are notably common in the North American population with prevalence of 2.5–3% of the population.^1^ The most common types of left‐sided VHD in the Western world are aortic stenosis (AS), aortic regurgitation (AR), and mitral valve regurgitation (MVR), with estimated prevalence of 0.4%, 0.5%, and 1.7%, respectively.^2^ Diagnosis and staging of VHD is primarily determined using Doppler echocardiography. However cardiac magnetic resonance imaging (MRI) is indicated in moderate or sever AR and chronic primary MR when images are suboptimal to assess heart volumes and function.^3^ Two-dimensional phase contrast MRI with single direction (through plane) velocity encoding is the standard-of-care technique to quantify blood flow but its capacity to assess complex valvular flow patters is limited.^4,5^ Four‐dimensional flow MRI (4 D flow MRI) is a novel imaging technique capable of measuring complex blood flow in the three principal directions and as a function of time, allowing for accurate quantification of blood flow in patients with VHD.^6,7^ A particular benefit of 4 D flow MRI is the retrospective selection of analysis planes at any location within the 3 D data volume to perform blood flow quantification, although analysis planes are typically static and don’t follow the motion of the heart valves. Valve tracking have showed to improve the characterization and quantification of eccentric regurgitation using 4 D flow MRI.^8^ Similarly, previous work demonstrated that machine learning (ML) can help to identify valvular dysfunctions and therefore used for the diagnosis of heart valve degradation.^9^ However, it requires further validation.

We hypothesize that ML when applied to dynamic valve tracking may improve the assessment of valvular blood flow in 4 D flow MRI datasets. In this study, we aimed: a) to assess the performance of dynamic valve tracking (assisted with ML for valve location identification) to quantify mitral and aortic flow, peak velocity and regurgitant fraction from 4 D flow MRI datasets; b) to compare dynamic valve tracking quantification with static analysis planes placed at aortic and mitral valve locations within the 4 D flow MRI datasets; and c) to evaluate intra- and inter-observer variability between both methods.

## Methods

### Patients, setting and study design

#### Study population

A total of 116 subjects were identified and enrolled at the time of clinical referral for cardiac MRI or research exam. Study cohort included 106 subjects, *n* = 19 with mitral disease patients (48 ± 18 years, 8 female), *n* = 65 with aortic disease patients (46 ± 15 years, 15 female) and *n* = 22 healthy controls (40 ± 13 years, 10 female). Patients were recruited under an a-priori sub-study of the Cardiovascular Imaging Registry of Calgary (CIROC, REB13-0902), a prospective observational registry at the Libin Cardiovascular Institute, University of Calgary. Patients were required to have confirmed aortic and mitral valve disease by trans-thoracic echocardiography or prior MRI, to be ≥18 years of age with not more than mild mitral valve insufficiency. Patients with any evidence of significant systolic dysfunction (left ventricle ejection fraction [LVEF] < 50%), history of known ischemic or non-ischemic cardiomyopathy, or complex congenital heart disease were excluded, as were patients with implantable devices or other recognized contraindications to MRI. Ten patients were excluded for poor image quality in the 4 D flow MRI datasets. Healthy control subjects were recruited and were required to have no known cardiovascular disease, hypertension, diabetes, renal disease or any standard contra-indications for MRI. Healthy control screening was performed by a certified nurse from our institution. The study was coordinated using Acuity® (Cohesic Inc., Calgary, Alberta) for the delivery of patient informed consent, health questionnaires and for collection of standard MRI-related variables. The study was approved by the institutional review board (IRB) at our institution and all subjects provided written informed consent. All research activities were performed in accordance with the Declaration of Helsinki.

#### Cardiac magnetic resonance imaging protocol

All healthy volunteers and patients underwent a standardized MR imaging protocol using a 3 T clinical scanner (Prisma or Skyra, Siemens Healthineers, Erlangen, Germany) inclusive of standard multi-planar steady-state free-precession (SSFP) cine imaging in four-chamber, three-chamber, two-chamber, at valve planimetry to characterize valve type, short-axis of the left ventricle (LV) at end-expiration, 3 D magnetic resonance angiography (MRA) of the thoracic aorta, and 4 D flow MRI.^10^ A trans-valvular jet in-plane velocity acquisition served as complementary velocity scout for 4 D flow MRI velocity encoding (V_enc_) selection. 4 D flow MRI was performed using a retrospectively triggered sequence (Siemens WIP 785 A) with respiratory navigator-based gating. Whole heart 4 D flow MRI was added at the end of the protocol for the assessment of 3 D hemodynamics. 4 D flow imaging parameters were: V_enc_ = 1.5–4.0 m/s, field of view = 200–420 mm × 248–368 mm, spatial resolution = 1.9–3.5 × 2.0–3.2 × 1.8–3.5 mm^3^, temporal resolution = 25–35 ms, phases = 30, and flip angle = 8–15°. Acquisition time ranged from 8–18 min.

#### 4D flow MRI analysis

**Figure 1.**
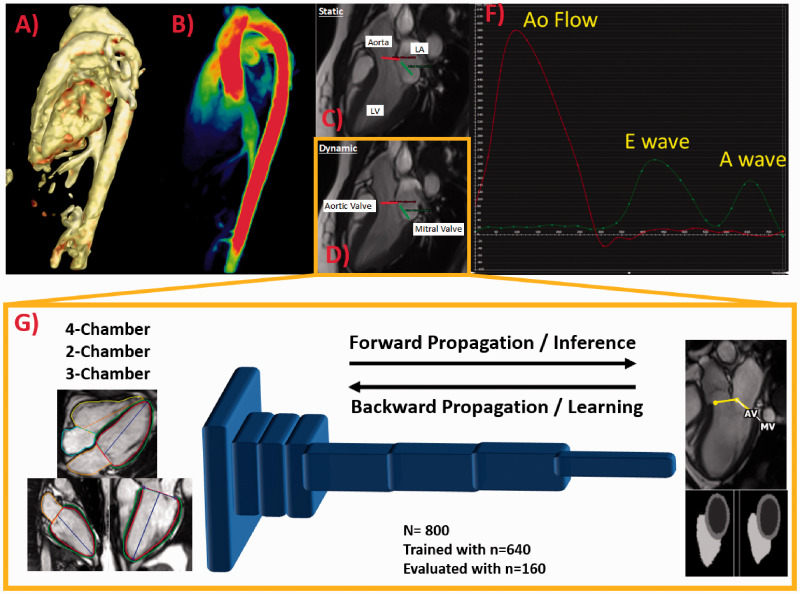
Workflow. (a) Shows pre-processed data and angiogram rendering; (b) shows velocity mapping; (c) illustrates static/manual plane positioning at aortic and mitral valve locations; (d) illustrates the automated machine learning detection of valve planes and tracking; (f) shows the aortic (red) and mitral (green) flow plots; and (g) shows the convolutional neural network used for segmentation of the left ventricle and detection of the aortic and mitral valve locations using two-, three-, and four-chamber images from the UK Biobank database (*n* = 800 total cases, trained with *n* = 640 cases and tested with *n* = 160 cases).

Cine MR images were processed and analyzed using cvi42 5.11.5 (Circle Cardiovascular Imaging Inc., Calgary, Canada) to determine LV end diastolic volume (LVEDV), and LV mass. Where appropriate, volume and mass measurements were indexed to body surface area (BSA), calculated using the Mosteller formula. Standard 2 D PC-MRI was used to provide conventional measures of hemodynamic significance, including flow volume, regurgitant fraction, and a peak velocity (PV) based on simplified Bernoulli’s equation. Aortic valve stenosis and regurgitation severity were ranged according to current guidelines.^1^ 4 D flow MRI analysis was performed using a prototype module from cvi42.^11,12^ Pre-processing corrections were applied to reduce noise, correct for eddy currents, and perform phase unwrapping in the case of velocity aliasing. ML module was trained for LV segmentation and valve location identification using publicly cardiac MRI data from the UK biobank.^13–15^ A total of 800 studies were selected, 640 studies were randomly included in the training process and 160 studies were used for evaluation as reported in a previous study.^15^ In our study the trained module was used to identify the location of the aortic and mitral valve in a 3-chamber cine. Tissue feature tracking was applied in the three-chamber cine series to create dynamic planes following valve motion (dynamic valve plane tracking).^16^ Then plane locations were interpolated into the 4 D flow MRI dataset where an automated contour detection was use for facilitating flow/velocity quantification (Figure 1). Contours were manually verified and corrected as needed. Net flow, regurgitant fraction, and PV were automatically calculated at each plane. A sub-cohort of 16 cases were assessed by two observers for assessing agreement, repeatability and scan-rescan variability. Automatic contour detection was used to quantify flow for each time point at every plane.

### Statistical analysis

Histograms and Shapiro-Wilks normality test were conducted to determine the distribution of the parameters in each cohort. Analysis of variance (ANOVA) or non-parametric equivalent was applied to compare control, aortic and mitral cohorts. Individual cohort comparisons were assessed by t-test or equivalent non-parametric test. Pearson’s correlation coefficients were calculated to identify relationships between static and dynamic measurements for net flow, regurgitant fraction, and PV. Observer repeatability and scan-rescan variability were evaluated by Bland-Altman analysis. For all statistical tests, a p-value of less than 0.05 was considered significant. All statistics were performed in SPSS 25 (Chicago, IL).

## Results

Subjects in control cohort were younger than aortic and mitral patients which showed similar age range (*p* = 0.015). Left atrial indexed volume was increased in mitral patients as compared with controls and aortic valve disease patients (*p* < 0.001). Table 1 summarizes demographic and cardiac MRI measurements.

**Table 1.** Demographic and cardiac MRI measurements.

Parameters	Control	Mitral	Aortic	*p*-values
Age (years)	37 ± 14	48 ± 17	48 ± 16	0.015
Sex, female (%)	1 ± 0.4	1 ± 1	1 ± 0.4	0.339
Height (m)	73 ± 84	36 ± 69	77 ± 88	0.179
Weight (kg)	80 ± 24	73 ± 16	85 ± 21	0.094
Body surface area	2 ± 0.3	2 ± 0.2	2 ± 0.2	0.066
Heart rate (bpm)	63 ± 10	65 ± 13	63 ± 12	0.786
SBP (mmHg)	113 ± 16	113 ± 15	110 ± 15	0.834
DBP (mmHg)	61 ± 16	66 ± 12	65 ± 13	0.652
LVEDV (ml)	166 ± 40	191 ± 49	177 ± 71	0.436
LVEDV indexed (ml/m^2^)	86 ± 17	102 ± 22	88 ± 32	0.096
LVEF (%)	62 ± 5	60 ± 9	61 ± 13	0.803
LVESV (ml)	64 ± 19	77 ± 29	72 ± 39	0.472
LVESV indexed (ml/m^2^)	37 ± 16	44 ± 18	36 ± 19	0.282
LV mass (g)	103 ± 32	109 ± 28	119 ± 57	0.389
LV mass indexed (g/m^2^)	52 ± 10	58 ± 11	59 ± 26	0.47
LVCO (l/min)	7 ± 2	7 ± 2	7 ± 3	0.742
LA volume (ml)	70 ± 16	98 ± 30	71 ± 27	0.013
LA volume indexed (ml/m^2^)	37 ± 9	53 ± 16	35 ± 13	<0.001

Overall subjects, aortic total flow was higher in aortic valve disease as compared with mitral valve disease (*p* < 0.05). Aortic regurgitation fraction was elevated in aortic valve disease subjects as compared with controls and mitral valve disease patients (*p* < 0.05). Mitral peak velocity was higher in mitral valve disease as compared with controls and aortic valve disease (*p* = 0.001). Similarly, mitral regurgitant fraction was higher in mitral patients (*p* < 0.05). A summary of aortic and mitral plane measurements and ANOVA test is provided in Table 2.

**Table 2.** Aortic and mitral valve measurements.

Parameter	Control (*n* = 22)	Mitral (*n* = 19)	Aortic (*n* = 65)	*p*-values
Aortic Total Flow (ml)	73 ± 19	65 ± 12	80 ± 23	0.025
Aortic Peak Velocity (cm/s)	117 ± 24	118 ± 76	133 ± 62	0.445
Aortic Regurgitant Fraction (%)	0.3 ± 0.4	4 ± 6	15 ± 17	0.003
Mitral total flow (ml)	65 ± 16	62 ± 17	66 ± 21	0.706
Mitral Peak Velocity (cm/s)	71 ± 19	109 ± 58	74 ± 32	0.001
Mitral Regurgitation Fraction (%)	–	12 ± 3	3 ± 1	0.032

**Figure 2.**
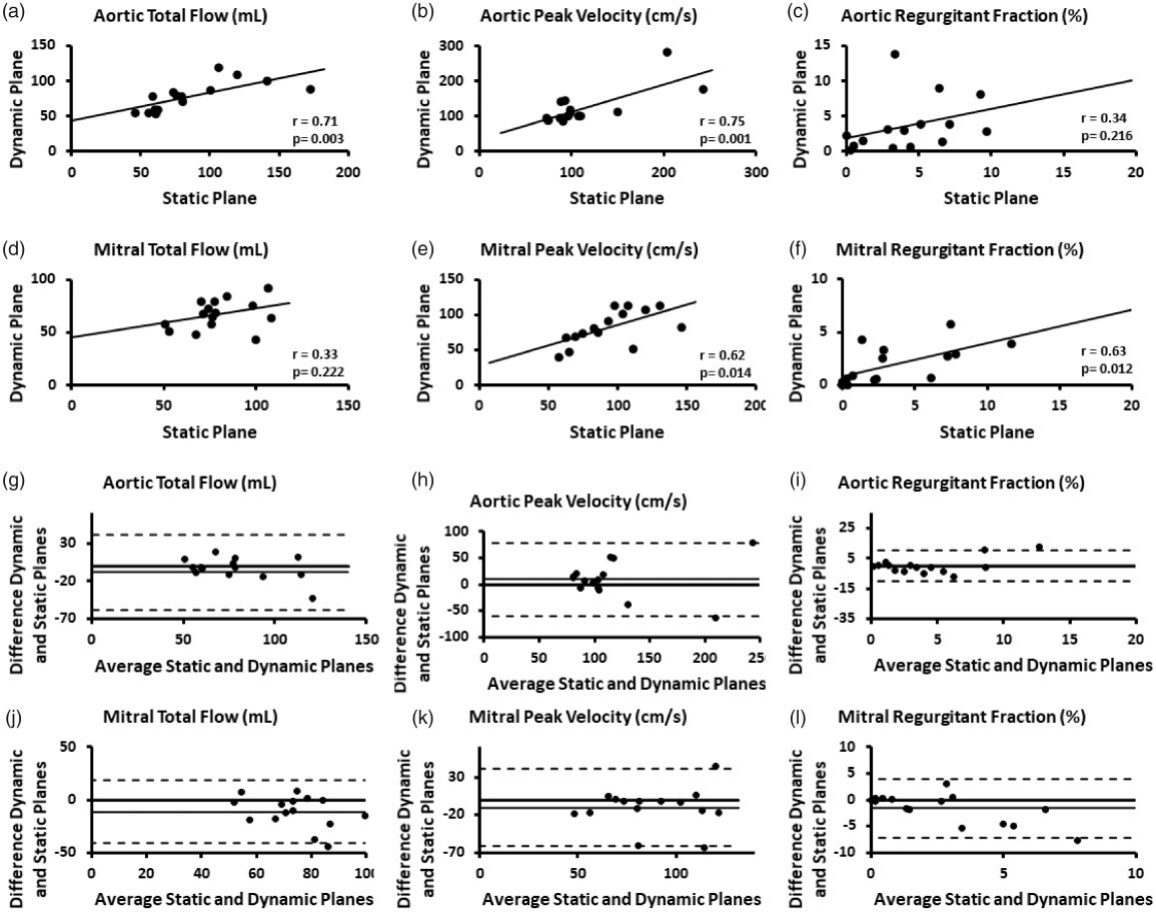
Static and dynamic planes correlation and agreement for aortic and mitral planes.

Static and dynamic planes in the aorta showed a good correlation for aortic total flow (*r* = 0.71, *p* = 0.003) and aortic peak velocity (*r* = 0.75, *p* = 0.001), mitral peak velocity (*r* = 0.62, *p* = 0.014) and mitral regurgitation fraction (*r* = 0.63, *p* = 0.012). Modest associations were found for aortic regurgitation fraction (*r* = 0.34, *p* = 0.216) and mitral total flow (*r* = 0.33, *p* = 0.222). Correlation plots are presented in Figure 2(a) to (f). Bias agreement between static and dynamic planes was greater in mitral measurements than in aortic measurements, as showed in Bland-Altman plots in Figure 2(g) to (l).

**Figure 3.**
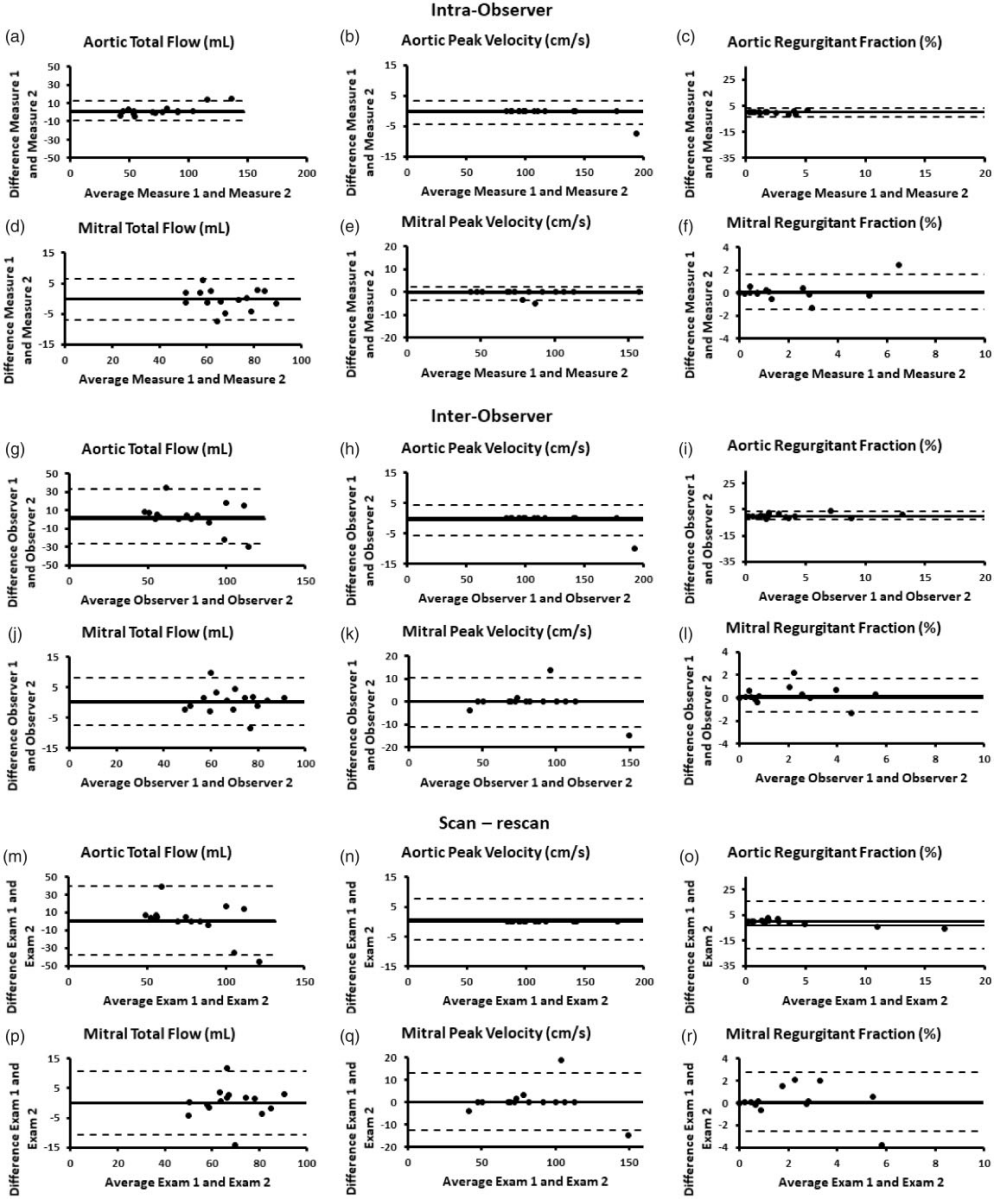
Intra-observer, inter-observer, and scan-rescan assessment for aortic and mitral planes.

Intra- and inter-observer variability showed good agreement for both aortic and mitral plane measurements. Measurements were more spread in the mitral plane. Intra-observer Bland-Altman plots are shown in Figure 3(a) to (f) and inter-observer plots in Figure 3(g) to (l). Scan and rescan measurements showed minimal bias for all cases. Aortic total flow, and mitral measurements showed larger spread in Bland-Alman plots (Figure 3(m) to (r)).

## Discussion

In this study, we demonstrated the feasibility of assisted aortic and mitral valve planning and tracking using machine learning in 4 D flow MRI datasets. Mitral measurements demonstrated more variability in the assessed sub-cohort. Despite the latter, the proposed approach demonstrated a good inter- and intra-observer variability, as well as scan-rescan variability.

### Comparison of results to previous studies

Doppler-echocardiographic indices (i.e. PV and valve orifice area) can be inconsistent or may be incongruent with the patient’s clinical status.^17–19^ For this reason, there has been interest in exploring innovative imaging modalities to help better identify patients who will benefit most from surgical intervention. 4 D flow-derived measurements found in our cohort were generally in fair agreement with findings in other MRI studies. While studying the mitral and tricuspid valve blood flow, Westenberg et al. found that the use of 4 D flow MRI with valve tracking to measure net flows over the mitral valve (MV) and tricuspid valve (TV) has been associated with markedly higher correlations between values than the 2 D PC-MRI (Pearson’s *r* = 0.34, *p =* 0.34 for 2 D PC-MRI as opposed to *r* = 0.91, *p* < 0.01 for 4 D flow MRI).^20^ In another study by Ewe et al. found that 4 D flow MRI and 3 D TTE can capture regurgitation better than 2 D TTE because they are not limited by geometrical assumptions and suboptimal alignment with the flow jet (*r* = 0.66, *p* = 0.005).^21^ Also, recent large-scale studies found MRI-derived regurgitant volume to be a better predictor of referral for surgery and all cause mortality than echocardiographic parameters.^22,23^ These findings may evoke changes in the diagnostic and prognostic workup of MV patients, causing MRI to gain ground in the clinical management of these patients. Our study showed that assisted valve planning and tracking can provide reliable clinical flow measurements.

### Study limitations

The analyst was blinded to comparative 2 D measures during processing of 4 D flow data. However, the analyst was not blinded to whether the subject was a healthy volunteer or patient with mitral or aortic valve disease at the time of cvi42 analysis. This may have introduced unintentional bias to this study and its results. Furthermore, the discrete spatial and temporal resolution of 4 D flow MRI may result in a systematic underestimation of peak velocity,^24,25^ due to partial volumes effects and temporal filtering when using only analysis planes instead of full volume analysis. This limits the accuracy of 4 D flow-derived parameters which may be underestimated. Turbulence and complex flow can also result in signal dephasing, which may further compromise the accuracy of measurements estimated from 4 D flow and 2 D PC-MRI.^26^

## Conclusions

Dynamic valve plane tracking assisted by machine learning showed good feasibility and performance for the assessment of aortic and mitral total flow, peak velocity and regurgitation fraction. Further, investigation of mitral and aortic severity quantification/grading and its association with valve disease is warranted.
